# They Told Me “This Isn’t a Hotel”: Young People’s Experiences and Perceptions of Care When Presenting to the Emergency Department with Suicide-Related Behaviour

**DOI:** 10.3390/ijerph19031377

**Published:** 2022-01-26

**Authors:** Jacinta Freeman, Penelope Strauss, Sharynne Hamilton, Charlotte Pugh, Katherine Browne, Suzanne Caren, Chris Harris, Lyn Millett, Warwick Smith, Ashleigh Lin

**Affiliations:** 1 Telethon Kids Institute, 15 Hospital Avenue, Nedlands, WA 6009, Australia; penelope.strauss@telethonkids.org.au (P.S.); sharynne.hamilton@telethonkids.org.au (S.H.); charlotte.pugh@telethonkids.org.au (C.P.); Ashleigh.Lin@telethonkids.org.au (A.L.); 2 Centre for Child Health Research, University of Western Australia, 35 Stirling Highway, Nedlands, WA 6009, Australia; 3 Commissioner for Children and Young People Western Australia, 469 Wellington Street, Perth, WA 6000, Australia; katherine.browne@ccyp.wa.gov.au; 4 Headspace, 64 Morrison Road, Midland, WA 6056, Australia; suzanne.caren@headspacemidland.com.au; 5 Mineral Resources Limited, 1 Sleat Road, Applecross, WA 6153, Australia; chris.harris@mrl.com.au; 6 Australian Childhood Foundation, P.O. Box 3485, East Perth, WA 6892, Australia; lmillett@childhood.org.au; 7 Cochair Youth Mental Health Subnetwork, QEII Medical Centre, Nedlands, WA 6009, Australia; warwick.smith@health.wa.gov.au

**Keywords:** suicidal behaviour, emergency department young people, experiences

## Abstract

In Australia, the number of young people presenting to the emergency department with mental health concerns, in particular, suicidal behaviour (defined here as suicidal ideation, thoughts, intent and attempts) is increasing. Little is known about the experiences of Australian young people who present to hospital emergency departments with suicidal behaviour. In this qualitative study, we conducted a series of focus groups with 55 young people aged 16–25 years, with a view to developing a framework for youth suicide prevention for Western Australia. The data were analysed using a general inductive analysis approach. We explored the experiences and perceptions of the care and management of 35 young people presenting to Western Australian hospital emergency departments. Participants described a range of negative experiences relating to the emergency department environment, staff attitudes and their treatment by staff. We argue that adapting ED practices and approaches to young people presenting with suicidal thoughts and behaviours based on these findings will result in lower rates of repeated presentations and admissions to hospital and lower rates of suicide attempts and deaths by suicide.

## 1. Introduction

Emergency departments (EDs) play a vital role in treating mental illness. People seek mental-health-related services in EDs for a variety of reasons, often as an initial point of contact for their mental health concerns or for after-hours care [1]. In Australia, the number of young people with mental health presentations to the ED is increasing, the highest rate being for those aged 18–24 years compared to other age groups (197 per 10,000 population) [2,3]. (The most common age group seeking help with acute mental health conditions from EDs is 15–24 year olds (22%) [4], consistent with most mental disorders emerging by the age of 25 years (62.5%) [5,6]. In Western Australia (WA), where this study was undertaken, a quarter of all attendances to EDs for mental health concerns between 2013 and 2015 were made by young people aged under 23, with suicidal behaviour being the number one reason for ED attendance in young people aged 15–24 [7]. A significant increase was reported for young people aged 13–17 presenting with self-harm, suicide risk and attempted suicide to Perth metropolitan EDs over the past decade [8]. Similarly, in New South Wales (NSW), the 15-to 19-year-old age group had the highest mental health presentation rates, most notable for those aged 10–14 years [9]. Young people aged 10–19 with suicide ideation commonly present to the ED, and this number is increasing, on average, by a staggering 27% annually [9].

Suicide is the leading cause of death among young people in Australia, and rates of youth deaths by suicide have also steadily increased over the past 10 years [10]. For young people aged under 14 years, suicide is rarely reported, but these figures have also increased over the past 10 years [10]. Between 2014 and 2018, WA had the third highest rate of suicide in the country in young people (aged 5–17 years), and the number who have died by suicide has doubled from 9 in 2009–2010 to 18 in 2018–2019 [11]). There has also been an increase in government inquiries investigating youth suicide in Western Australia [12,13,14] Among the general population, those who are at risk of suicide frequently attend the ED and are at greater risk of dying by suicide immediately after discharge or within a year following their presentation to the ED [15,16]. The period following a first suicide attempt is associated with the highest risk of further suicide attempts [17] and deaths by suicide [17,18,19,20]. Thus, optimal treatment at presentation to the ED provides an important opportunity for intervention and suicide prevention.

Scholarly exploration of the experiences of adults presenting to the ED has shown that labelling, stereotyping, stigmatisation, insufficient help and poor attitudes of ED staff were common [21,22]. Following a suicide attempt, patients have also identified low levels of satisfaction with ED care [23,24,25]. There has been less exploration of the experiences of young people who present to the ED with mental health concerns or suicidal behaviour. A number of studies have shown that despite the existence of guidelines for best practice for responding to suicidal behaviour and aftercare in the ED, many young people experience poor care and negative staff attitudes [23,25,26,27]. Moreover, they are often turned away without follow-up support [26,28,29]. These negative experiences can have repercussions that impact help-seeking for subsequent suicidal ideation and suicide attempts [17,23,27] (and can increase the likelihood of future suicide attempts and fatalities [21,23,27].

There are multiple reasons why young people with mental health concerns choose to attend an ED, including that they feel a strong need for urgent care or consider that an ED can offer access to better or faster care [30,31]. However, the management of clients with a mental illness in the ED is complex and challenging. Inconsistencies in mental health triage and delayed access to mental health clinicians have been reported as concerns. EDs may not always be equipped for mental-health-related presentations, and often staff lack the skills and confidence to deal with suicide-related presentations and are not trained to support people in need of mental health care [32]. Because of these concerns, there is a need to formally recognise those presenting to the ED with suicide ideation as a priority population [33].

The views of young people who have presented to the ED with suicidal behaviour (defined here as suicidal ideation, thoughts, intent and attempts) in Australia are currently underinvestigated. In this article, we contribute to reducing to this gap in knowledge by identifying the perspectives of young people presenting to the ED with mental health concerns. We describe young people’s experiences of the ED when presenting with suicidal behaviour, including their reasons for attending the ED, their experience upon arrival to the ED, their perception of staff attitudes, their perceptions of care during their stay and their experiences of discharge from the ED.

## 2. Method

### 2.1. Participants

The data presented here were from a project that was developed to inform a youth suicide prevention framework for Western Australia. The broader project described what works well and what does not work well for youth suicide prevention from the perspectives of two populations: (i) young people aged 16 to 25 years who had ever accessed mental health support or participated in suicide prevention training; and (ii) professionals working in the youth mental health sector [34]. A total of 55 young people aged between 16 and 25 years took part in the focus groups. The cultural diversity reflected the diversity of the Perth metropolitan area. As an example, Aboriginal young people were slightly overrepresented in the sample (Table 1). Data from the young people who indicated an experience in accessing the ED for suicidal behaviour were reported in this paper. In total, 35 young people from the sample of 55, described having an experience of visiting an ED for suicidal behaviour—the data regarding these cases is reported here.

### 2.2. Recruitment

Young people aged 16 to 25 years who had experience with a mental health service provider, had participated in any suicide prevention training programs or had an interest in youth mental health and suicide prevention were invited to participate in the project. Researchers recruited participants for the focus groups through professional networks, stakeholder organisations, services providing crisis and transitional supported accommodation, services providing alcohol and drug detoxification, youth mental health service providers, the Youth Affairs Council of Western Australia (YACWA) and a local resource centre for LGBTIQ+ young people.

### 2.3. Data Collection

Eleven focus groups (nine in the Perth metropolitan area and two in a regional town located in the southwest region of WA) were conducted in 2019. The focus groups had between two and nine participants and varied in length of time from approximately 45 to 90 min. The facilitators used open-ended questions to guide the discussion. The focus groups were held onsite at the host organisations. A focus group that was hosted by an Aboriginal organisation was facilitated by an Aboriginal and a non-Aboriginal researcher to provide cultural safety. The other 10 focus groups were facilitated by the same two researchers (J.F. and C.P.). J.F. is a project manager, and C.P. was a provisional psychologist at the time. Both had completed the Applied Suicide Intervention Skills Training.

Prior to the commencement of the focus groups, young people reviewed the information sheet and completed a written consent form. Participants were also encouraged to complete a de-identified form that included demographic questions and questions concerning experiences with justice, child protection, cultural communities and suicidal behaviours, as reported in Table 1.

Young people in the focus groups presented their views and perspectives on how youth suicide prevention can be improved for WA as part of the larger project. Part of the focus group discussion presented here was focused on young people reporting their experiences of the ED and their perceptions of care and treatment when they had presented to the ED with suicidal behaviour.

All focus groups were audio-recorded, and notes were taken during and immediately after by the researchers running the focus group to ensure a high level of rigor.

### 2.4. Ethical Considerations

Ethics approval was granted by the University of Western Australia Human Ethics Research Committee (RA/20/4850). The researchers acknowledged the sensitivity of the research topic; thus, participants could not be actively suicidal or under the influence of drugs or alcohol. If the young person participating in the focus groups was residing in crisis accommodation, attending drug and/or alcohol detoxification or a member of a mental health service youth reference group, they were assessed by the organisation for their appropriateness to participate in the focus group based on the young person’s mental health at the time. On the day of the scheduled focus group, it was again confirmed with their case manager from the host organisation that the young person was currently mentally healthy and able to safely participate in the focus group. For safety reasons, some focus groups were attended by a staff member from the host organisation who was more familiar with the participants’ cues for whether and/or when they became upset or distressed.

### 2.5. Data Analysis

The data were analysed using a general inductive approach to thematic analysis, providing an authentic approach for drawing findings based on observation rather than testing a priori hypotheses [35]. The focus groups were audio-recorded and transcribed verbatim. All potentially identifying information was removed from the transcripts. Clarification was sought out by the facilitators during the focus groups by repeating the general responses back to participants and checked against any notes taken by facilitators during the focus groups. Data analyses were undertaken by J.F. and C.P. using the following steps as suggested by [36]. They (a) read and familiarised themselves with all the textual material and documented points of interest, (b) met several times to compare notes and agreed on a set of initial codes, (c) developed a list of potential themes based upon the initial set of codes using NVivo qualitative data analysis software (Version 12, QRS International Pty Ltd., Melbourne, Australia), (d) reviewed and checked whether the themes worked in relation to the coded extracts that were used to sort units of data into meaningful categories, (e) defined and named the themes with ongoing analysis in an iterative process to refine the specifics of each theme and the overall story that the analysis conveys and (f) wrote the summary of experiences.

## 3. Results

A total of 55 young people took part in 11 focus groups. The views of 35 participants who voiced their own experiences or those of a friend who had attended an ED with suicidal behaviour are represented in the threads presented in this paper. Demographics, incidence of suicidal behaviour, engagement with government systems and other lived experience of 53 young people who participated in the focus groups are described in Table 1. The demographic data collected were not linked to the responses of the 35 participants who shared their experiences for this paper at the time of data collection. The missing data are due to two participants declining to complete the demographics and lived experience form.

The findings presented here reflect the steps taken by a young person as they enter an ED and then progress through the department. These are (1) the pathway to the ED for a young person with suicidal behaviour; (2) attendance at the ED (which includes the triage and treatment areas of the ED) and (3) discharge from the ED. Key themes were identified within each of these categories and structured around the processes of the ED and the young people’s experiences of the ED based upon their perception of ED staff attitudes. The main subthemes within each of these themes are discussed below.

### 3.1. Pathways to the ED for Young People with Suicidal Behaviour

In this study, most participants with risk for suicide, suicidal behaviour or a suicide attempt reported that attending the ED at a hospital was one of their help-seeking approaches. One participant said,

“I’ve just taken a step forward; I’ve tried to get help.”

Most participants who had experienced suicidal behaviour and attended the ED described how and why they ended up at the ED. A number of them reported being taken to the ED by emergency services. As described by participants,

“I had an argument with my mum one time and tried to commit suicide, and the police ended up taking me to the hospital.”

“I rang a [mental health emergency phone number] when I was living in a different hostel and they basically said that I was attention-seeking and that I didn’t need to go to hospital, but then 10 minutes later, the cops were at the hostel taking me to hospital.”

Participants also reported being taken there by a family member.

“I know like when my nana took me to the A&E, if it (suicidal behaviour) gets really bad then, off to the hospital, yeah…”

“They (parents) just rushed me to ED—they pushed me to go into the inpatient mental health ward at the hospital.”

Some young people reported self-presentation. Participants described their repeated attempts to get assistance as follows:

“I’ve been there hundreds of times asking for help.”

“Uhm, yeah, I’ve been in the emergency department for—after suicide attempts probably for that. But I don’t know how many times now.”

- Young People’s Views on Choice and Lack of Alternatives to the ED

The ED was described by the participants as the only option available to them when experiencing suicidal behaviour and seeking help. They said there should be an alternative to the ED when they were feeling suicidal. As described by one participant,

“I didn’t really get a choice because I was quite young when I presented to a teacher with suicidal ideation.”

The lack of alternatives to the ED was also reported by participants who were taken to the ED either by family, friends, the police or ambulance.

“…but there’s no other option, so it’s like when you reach that point or something hasn’t been noticed earlier, it’s like that is the only option.”

Another participant described the lack of suitability of attending the ED when experiencing suicidal behaviour as follows:

“I think, I think they need a complete new system rather than ED ‘cause it’s not appropriate like place for anyone to be taken but it is the only option. So, it’s either ED, stay at home, and that’s not helping at all go to ED because that is the only thing, only place to go.”

Only one participant stated that the ED was part of their crisis plan because of a lack of alternative support:

“So sometimes, like, for me, I don’t tell people when I’m suicidal and I’ll just end up acting on it, but sometimes I, like, when I know I definitely do need help, I do go to a hospital ‘cause I have a crisis plan.”

One young person who had presented to the ED with suicidal behaviour on multiple occasions stated that they felt they had no other options, and going to the ED was their one and only help-seeking strategy:

“That’s a really bad system for like when you’re in crisis, the only option is ED.”

### 3.2. Attendance at the ED for Young People with Suicidal Behaviour

Participants overwhelmingly described negative experiences when attending the ED with suicidal behaviour, consistent with previous literature [22,23,27]. They perceived the staff as judgemental and dismissive upon their arrival. Most young people’s negative experiences were based upon staff attitudes, stigmatisation of mental health difficulties and exclusion.

#### 3.2.1. Experiences on Presentation to the ED

Participants expressed that their reception, treatment and care was dependent upon their mode of arrival (self-presenting compared to arriving by ambulance) and their suicidal behaviour (suicidal thoughts compared to a suicide attempt). They described being taken more seriously by ED staff if they had arrived with police or in an ambulance, and/or if they had attempted suicide as compared to feeling suicidal:

“Yeah, and, like, if you come in by police, they take you more seriously. If you get taken in by ambulance, they take you more seriously.”

Stigmatising attitudes towards suicidality has been shown to be a barrier to seeking professional help [37]). The stigma associated with mental health difficulties, especially suicidal behaviour, and the inability to be able to disclose symptoms privately resulted in these young participants being afraid to present to the ED. Young people reported that they did not want other members of the public knowing their reasons for attending the ED. This is difficult as triage is prominently located at the entrance of the hospital in front of the waiting room. These participants captured the need for privacy as follows:

“I feel like they should have like a separate room because no one wants to know, we don’t want other people to know why we’re in there.”

“…a side room that you can just go into and say, ‘I’m here because of this thing,’ (suicidality) and then go back out and like—or something just so that you don’t have to say in front of everyone, ‘Hey this thing.’ (suicidality/suicide related behaviour).”

Limited privacy in the triage process meant young people found it difficult to disclose suicidal feelings in a safe way. For young people living in regional areas, the lack of anonymity and privacy was increased. One young person reported that they required a support person to assist them to disclose their feelings to the triage nurse. They stated,

“I made [name] speak for me when he took me to the emergency department and made him tell, tell them what was going on.”

“Uhm, like when I did go in the emergency, I found it was, was really scary having to talk through that thin slit, you know, without shouting and then telling everybody else in the emergency why, why I was there—I need help.”

#### 3.2.2. Young People’s Experiences of Staff Attitudes

Experiences of the ED can be overwhelmingly negative, and staff can be dismissive, disinterested and lacking in knowledge [38]. This was reiterated by the young people in this study, especially those who had made repeat presentations to EDs. They described the ED staff as being rude, dismissive and judgemental. Participants reported that

“They judge you and the doctor—you go walking to ED and they’d go—and they—you’ve been there many times and they go, ‘You’re back again’.”

“All she wanted to do was just brush me off, get me out of the emergency department.”

Another young person received the following comment from an ED staff member:

“Well, you know, we can’t find a bed for you. I’m sorry, you’re gonna have to go because this isn’t a hotel.”

Many young people reported feeling like they were an inconvenience to ED staff and other people in the waiting room. They reported hearing staff make comments about their presentation and request for help, especially those who had attended the ED on multiple occasions with suicidal behaviour.

“It’s like you’re like an inconvenience, like sort of like, you know, just an annoyance and it’s like you’re always waiting hours just to be even seen.”

“They just say, ‘Oh, you again’.”

Participants reported observing people who appeared older and presented with an unintentional physical life-threatening injury were treated more seriously. They also reported that they thought ED staff did not see mental health (including suicidality) as a priority compared to non-intentional physical injuries or other physical health conditions. Participants described observing other people in the waiting room receiving assistance and care before they did, despite themselves waiting for longer periods of time. Young people described this as a form of invisibility:

“And to them it’s not really a crisis ‘cause they’ve people come in like dying or whatever but it’s not helpful to sit in the waiting room with no one to come in to see you or even really notice that you’re there, and then you wait.”

“I’ve waited one time over 20 h just to speak to the psych ‘cause some hospitals don’t have a 24 h psych liaison nurse, so then you wait all night, morning to be seen for like 20 min, and then given a crisis number and on your way and it’s like, ‘What have you helped me with at all?’”

“We’ve had some nurses in the emergency department, they’re pretty rude to me, but it’s probably because they’re dealing with a 16-year-old who is just crying in the middle night and—yeah, but they are not very nice sometimes. They just treat you like you’re stupid.”

Failing to recognise mental health issues as potentially life-threatening can have devastating consequences, as highlighted by one young person who described a friend’s experience of seeking help:

“…prior to his suicide, he did try and admit himself into two hospitals, but they wouldn’t take him because they just didn’t see it as being severe. He was saying that he felt like he would take his own life, that he would hurt himself, but they just didn’t think that it warranted him being in the hospital and obviously that then led to him taking his own life.”

### 3.3. Young People’s Experiences of Care upon Discharge from the ED

Young people described their experiences of care upon leaving the ED as generally inadequate. They voiced their concerns about the lack of support when leaving the ED, the level of support that they required in the community upon discharge, the expectation that other family members would take responsibility for their care in the community, and the potential consequences that they anticipated as a result of their perceived negative experience.

#### 3.3.1. Perceived Lack of Follow-Up Support in the Community after Leaving the ED

Young people reported that they were usually discharged because they felt that the ED staff considered them to be not unwell enough to remain in the ED or that they were no longer actively suicidal in the ED. Young people felt that ED staff did not take their suicidal behaviour seriously and thought that they would be safe once they were discharged from the ED.

“They don’t give you follow-up support. They just kind of expect you to go and be fine just because you’ve had a small like, 24 h break from something, they expect you to be okay.”

“When I left, when I left the hospital (after a suicide attempt), they kind of just said, ‘See you,’ it was as bad as that, and they didn’t provide me with any information or any follow-ups.”

Most young people explained that they either received no support or minimal follow-up from professional services in the community, despite feeling that they required ongoing support. Even if a brochure or crisis numbers were provided, young people reported that they did not necessarily have the resources or capacity to seek support for themselves following discharge.

“So, like, I know a couple of people that get sent home and end up trying to commit suicide because they don’t know who to turn to.”

“Uhm, it was the first time I’d had really major suicidal thoughts and, uhm, the staff in the hospital were really supportive in helping me work out what I was feeling and, uhm, some coping strategies and writing up my crisis plan, but then when I was, uh, discharged, I had one appointment with the community mental health and they cleared me and I haven’t heard from them since.”

“… there needs to be more follow ups, they need to make sure you have follow-up appointments, ‘cause they don’t. They just leave you to your own devices once you’ve left. They need to make sure you have those follow-up appointments to remain supported in the community once you’ve left emergency.”

#### 3.3.2. ED Staff Expected Young People to Have Family Support

Young people who lived with family members reported that the ED staff expected that their family would be responsible for their safety and care upon discharge. Nearly half of the participants in the focus groups had a parent or caregiver with a mental illness, and many felt that their parent/caregiver was not appropriate to be supporting them. They reported that they did not seek mental health support from their parents for fear of either upsetting their parent or worsening their parents’ own mental health issues.

“Both of my parents have mental health, so they understand mental health, but when it comes to suicide, they just don’t wanna know about it.”

“She (Mum) doesn’t support me at the moment, but she hopefully will. That’s all you can kind of ask for, is you hope they do eventually.”

#### 3.3.3. Young People’s Experiences in the Community after Discharge from ED

This lack of support in the community meant that the young people were returning to the ED with suicidal behaviour. Participants described a combination of lack of support in the community and the lack of options for support services for young people experiencing suicidal behaviour.

“Normally, I just end up back in hospital by, like, the cops or ambulances because, like, especially on a weekend, there is no support, really.”

“I end up just doing something because no one’s taken me seriously and, yeah, I end up back in hospital and they take me seriously ‘cause I’ve done something, yeah.”

A young person who was supporting a friend with suicidal behaviour said,

“She was taken to the hospital and back the next day, and it’s still going on.”

Young people shared how their initial experience in EDs informed their decisions regarding future help-seeking with suicidal behaviour. Those young people who described a negative experience in the ED reported that they may be deterred from attending the ED with suicidal behaviour in the future and/or were concerned that they might end up in a vicious cycle of attempting suicide and presenting to the ED.

“… definitely kind of making me not wanna go back there ‘cause first time it’s just like if it’s good then you wanna go back, if it’s not then kind of, you know, it’s 50/50 if you wanna go back, you know, ‘cause that wasn’t helpful for the first time, it’s not gonna be helpful the second time.”

“Discharge you and then you come back. And it just keeps going and going until like you either don’t need to go to hospital anymore or something bad happens.”

## 4. Discussion

This study is, to our knowledge, one of only a very few qualitative studies that have explored the experiences young people presenting with suicidal behaviour to the ED in Australia. Our focus group participants were a diverse cohort who recalled their journeys (or those of friends) through the ED experience, from the first decision to attend the ED, to receiving treatment and to finally being discharged. Participants’ experiences through all stages of the ED process were overwhelmingly negative. This aligns with other national and international research on experiences of presenting to the ED with suicidal thoughts and behaviour, although much of the literature is either not specific to youth, concerns self-harm presentations or had a combination of suicidal and non-suicidal intent [38,39,40,41,42]. We argue that young people with lived experience of attending the ED are uniquely qualified to contribute to ways of improving care for suicidal behaviour. Their experiences can inform efforts to minimise distressing, improve patient outcomes, reduce ED crowding and waiting times and reduce the likelihood of future episodes of suicidal behaviour.

The Australian emergency health care system is designed to deliver safe and effective health care to patients in a timely, cost-efficient manner. The ED plays a critical role in delivering emergency health care services, with young people with mental health concerns increasingly presenting to an ED both at a state and national level [2,4,8,43]. Increased demand for the ED more generally has been attributed to a number of factors: a growing elderly population [44], increasing population co-morbidities [45], reduced access to primary health care providers and after-hours care [46] and changes in health-seeking behaviours [47]. Most recently, in Australia, the COVID-19 pandemic has resulted in a reconfiguration of ED services, which has led to increasing levels of “ambulance ramping” [48,49]. These factors have led to increases in hospital and ED overcrowding, longer waiting periods, poorer patient outcomes [50] and patients leaving without having their concerns attended to [46,51]. These multiple and confounding factors have ramifications for young people presenting to EDs with suicidal behaviour.

Despite previous negative encounters, young people in this study described the ED as being the only option available for help with suicidal thoughts and behaviours. Other studies have found that the ED was seen as the only available choice for individuals with mental health concerns including suicidal behaviour, self-harm, substance misuse, anxiety and depression [52,53,54]. Alternatives to presenting to the ED for young people needs to be further explored to assist with reducing negative experiences and promoting help-seeking behaviour.

Young people recounted not being taken seriously and having to wait lengthy periods of times, sometimes up to 15 hours, before progressing to a suitable care environment. This is consistent with findings of two reports commissioned by the Australian College of Emergency Medicine [4,55]. The lengthy waiting periods exacerbated young people’s perception that their presentation was not considered as serious as physical health symptoms and that they were wasting staff’s time. Participants in this study reported that they felt that young people were an inconvenience to ED staff and that they were treated as though their mental health problems were insignificant [39,56].

In particular, the findings of this study were remarkably similar to a report that surveyed and interviewed adults who had been hospitalised for a suicide attempt [24]. They found low levels of satisfaction with the ED, poor staff attitudes towards patients and poor aftercare. The report found that patients and carers reported that their emotional distress was not attended to, with many believing they were discharged too rapidly and were left to seek their own options for ongoing care. Similarly, in the current study, young people disclosed that they were the recipients of dismissive and judgemental comments, ED staff made light of the severity of their symptoms, repeat presentations were negatively acknowledged and most young people stating that they were not ready for discharge at the time they were discharged. Experiencing the negative attitudes of staff within the ED can have repercussions on future help-seeking behaviour and puts young people at risk of further suicide attempts and death by suicide [18,23]. To improve ED experiences for young people, effective communication, empathetic care and humanising responses from ED staff are critical [46]. It has been reported that ED staff feel more confident in managing physical injuries and lack confidence in assessing and managing suicide-related presentations and, as a result, additional training for ED staff is required to increase their confidence when working with suicidal young people [57,58,59].

Similar to previous literature [23,60], young people in this study reported leaving the ED after presenting with suicidal ideation feeling unsupported in the community or that the level of support was insufficient for their needs. The ED presents an opportunity for suicide prevention as young people want (and need) to be supported by genuine and non-judgemental ED staff. There is a critical requirement to implement aftercare and community support for young people in order to reduce the rate of youth suicide [61].

### 4.1. Implications

Until there is an alternative to the ED for young people with suicidal behaviour, their experiences are unlikely to improve. Alternatives to the ED for people in mental health crisis are currently being trialled across several states in Australia and are derived from the United Kingdom’s successful Safe Haven café model [62]. At the very least, staff attitudes and competencies need to change to provide a more empathetic environment for highly distressed young people.

With increasing numbers of young people presenting to the ED with suicidal behaviour, follow-up care in the community is essential. Young people who have made a suicide attempt are at a greater risk of dying by suicide in the future. In particular, death by suicide is higher immediately after discharge from ED and within 12 months following a suicide attempt [15]. Individuals who present to a hospital with suicidal ideation are at risk of repeat presentations and future self-harm. Indeed, the period following a first suicide attempt is associated with the highest risk of a further suicide attempt [17,18,19,20]. There is a significant need to formally recognise young people presenting to hospital with suicide ideation as an important at-risk group and to see the ED as a vital setting for intervention. Young people may also be less likely to seek help for future suicidal attempts if they have had a previous experience of negative staff attitudes and unsatisfactory care. The reduced likelihood of seeking help for future suicidal behaviour results in young people remaining in the community with suicidal behaviour and being at greater risk of dying by suicide.

### 4.2. Strengths and Limitations

The main strength of this study was the lived-experience descriptions provided by the young people who shared their stories and experiences. Often young people with suicidality are excluded from research based on their apparent risk [63] The overrepresentation of Aboriginal participants was also valuable, given the higher youth suicide rates in Aboriginal young people [10,14,64]. However, the findings of this study may also not be reflective of the experiences in other EDs across Australia and internationally. Other limitations are related primarily to the sample, as participants were not representative of all young people with an experience of ED presentation for suicidal behaviour, as they had to be mentally well enough to safely take part in the discussion on suicide. There were more females than males and non-binary people in our study. Most of the participants resided in the Perth metropolitan area, with only two focus groups being held in a regional town. There are other limitations to this study, including the small number of participants, retrospective recall bias and the inability to perform member checking due to the protection of confidentiality through anonymity. The researchers were not able to determine who accessed a paediatric ED compared to an adult ED, which warrants further research. However, these limitations are difficult to avoid and should be considered in relation to the study’s aim, including discussing a potentially sensitive subject within a vulnerable group and identifying young people’s experience of ED and services that require improvement to reduce youth suicide.

## 5. Conclusions

This study addresses a gap in the literature by considering young people’s experiences of presenting to EDs with suicidal behaviour. The ED provides an opportunity for suicide prevention, but EDs in Western Australia are not currently meeting the needs or providing a safe space for young people presenting with suicidal behaviour. Support for young people after they have made a suicide attempt that has been informed by research evidence needs to be enhanced. ED staff attitudes towards young people with suicide-related presentations have the potential to be addressed through additional training. Best-practice guidelines have recently been established for suicide-related crisis response and aftercare in the ED [26].An integrated approach to care is required, with improved referral pathways and sharing of information between health care systems. It is essential that every person who leaves the ED feel safe and supported; it is vital that EDs are equipped to care for young people with suicidal behaviour. We propose that there is a need to explore the development of alternatives to the ED for young people seeking help, and until then, ED staff need to better support young people with suicidal behaviour. The current service delivery model of EDs is not a suitable environment for young people experiencing a mental health crisis.

## Figures and Tables

**Table 1:** Demographics and lived experiences of participants.

Demographics	N	%
Gender		
Non-binary	4	8%
Male	16	30%
Female	33	62%
Age		
Average age: 19.5 years		
Min age: 16 years		
Max age: 25 years		
Ethnicity		
Aboriginal	7	13%
Born in another country	10	20%
Born in Australia	34	67%
LGBTIQA+		
	17	32%
Refugee/migrant/culturally and linguistically diverse		
	8	15%
Refugee	2	4%
Living with a disability		
	7	13%
Experience of current and previous homelessness		
	22	42%
Experience of Youth Justice Systems/police		
	19	36%
Experience of out of home care *		
	13	25%
Experience as a child of a parent with a mental illness		
	26	49%
Have you ever experienced suicidal thought?		
Yes	41	77%
Have you ever made a suicide attempt/s?		
Yes	24	45%

* These figures presented may be underrepresented as the term out of home care (OOHC) was used by the facilitators in the focus groups; some young people were unfamiliar with this term and asked for clarification (despite having experience of OOHC) and used the terminology “DCP” (Department of Child Protection).

## Data Availability

The data is stored securely onsite at Telethon Kids and we have elected not to have it publicly available due to the sensitive nature if the study and the data is qualitative.
